# Natural Cyclopeptide RA-XII, a New Autophagy Inhibitor, Suppresses Protective Autophagy for Enhancing Apoptosis through AMPK/mTOR/P70S6K Pathways in HepG2 Cells

**DOI:** 10.3390/molecules22111934

**Published:** 2017-11-11

**Authors:** Lihua Song, Zhe Wang, Yurong Wang, Di Guo, Jianhong Yang, Lijuan Chen, Ninghua Tan

**Affiliations:** 1 School of Traditional Chinese Pharmacy, China Pharmaceutical University, 639 Longmian Avenue, Nanjing 211198, China; songlihua4835@163.com (L.S.); wangzhe153807105@163.com (Z.W.); yurong1987213@163.com (Y.W.); guodi33@163.com (D.G.); 2 State Key Laboratory of Biotherapy, Sichuan University and Collaborative Innovation Center for Biotherapy, Chengdu 610041, China; yjh0742043024@hotmail.com (J.Y.); ljchen@scu.edu.cn (L.C.); 3 State Key Laboratory of Phytochemistry and Plant Resources in West China, Kunming Institute of Botany, Chinese Academy of Sciences, Kunming 650201, China

**Keywords:** RA-XII, cyclopeptide, autophagy, apoptosis, mTOR, HepG2 liver cancer cells

## Abstract

Liver cancer is a progressive, irreversible and aggressive malignant disease, which has no effective chemotherapeutic drugs. RA-XII, a natural cyclopeptide isolated from the traditional Chinese medicine *Rubia yunnanensis*, exerts anti-cancer and anti-inflammatory activities. This work aimed to investigate the effects of RA-XII on a hepatic tumor and its underlying mechanisms in human hepatoma HepG2 cells. The results showed that RA-XII effectively inhibited the proliferation of HepG2 cells. Consistently, RA-XII significantly induced apoptosis in HepG2 cells by decreasing the expression of caspase 3, 8, 9, and promoting the Cleavage of PARP. Moreover, RA-XII-induced apoptosis was attenuated in the presence of apoptosis inhibitor N-Benzyloxycarbonyl-Val-Ala-Asp (O-Me) fluoromethyl keton, suggesting that RA-XII induced apoptosis-cell-death in HepG2 cells. Furthermore, autophagy-related proteins and mRNA levels were dramatically reduced after RA-XII treatment. Meanwhile, we observed that autophagy inhibitor chloroquine could enhance apoptosis in RA-XII-treated HepG2 cells, indicating that autophagy played a protective role in HepG2 cells and RA-XII might inhibit protective autophagy. Further analysis showed that RA-XII inhibited AMPK phosphorylation and led to the mTOR/P70S6K pathway activation, suggesting that RA-XII inhibited autophagy through AMPK/mTOR/P70S6K pathways. This study demonstrated that RA-XII promoted apoptosis and inhibited protective autophagy through AMPK/mTOR/P70S6K pathways in HepG2 cells. In conclusion, these findings suggest that RA-XII might potentially be a candidate as an autophagy inhibitor agent for further therapy of liver cancer.

## 1. Introduction

Liver cancer is one of the leading cancers and the third cancer-related cause of death, and has been one of the few cancers with a rapid upward trend all over the world [1]. The high mortality rate of liver cancer patients is due to the difficulty in diagnosing at its initiation, its rapid progression, and its resistance to therapy [2]. A tremendous amount of effort has been put into developing more credible biomarkers for liver cancer detection and novel targeted chemotherapeutic agents [3,4]. Consequently, it is necessary to explore more efficacious therapies and prevention strategies for liver cancer.

Apoptosis and autophagy are two types of programmed cell death [5]. Apoptosis is traditionally considered to be a major mechanism to kill tumor cells. Two signaling pathways named extrinsic and intrinsic pathways regulate the cell apoptosis, in which caspase activation is involved. Caspase-9 is the initiator caspase in mitochondria-mediated intrinsic pathway and caspase-8 is the initiator caspases in death receptor-mediated extrinsic pathway. After initiator caspases are activated by cell stress or signals from other cells, executioner caspases are activated to kill the cell by degrading proteins indiscriminately [6]. However, autophagy can promote or suppress tumor cell survival. On one hand, continuous stress and excessive autophagy could lead to autophagic cell death [7]. On the other hand, autophagy enables cancer cells to survive under hostile stress including starvation, hypoxia and drugs [8,9]. Autophagy, an intracellular degradation pathway, is a complex catabolic process that involves the recycling and breakdown of long-lived proteins and useless intracellular organelles within cells. To maintain their rapid proliferation and differentiation, cancer cells require plentiful oxygen and energy during progression and invasion. As a key regulator for cellular homeostasis, autophagy is related to the survival of tumor cells and plays a cytoprotective role in cancer cells [10,11,12]. Therefore, targeting the autophagy process in liver cancer may provide new approaches to liver cancer treatment and prevention [13]. There are substantial evidences to show that autophagy inhibition prohibits the pro-survival effects of autophagy and enhances cytotoxicity in combination with anti-cancer therapies [11,14]. It has been reported that autophagy inhibitors can potentiate the efficacy of carfilzomib [15], cisplatin [16], celecoxib [17], paclitaxel [18], and anthocyanin [19] in cancer cells. Accordingly, it will appeal to explore new drugs with inducing apoptosis and inhibiting autophagy for liver cancer therapy.

**Figure 1:**
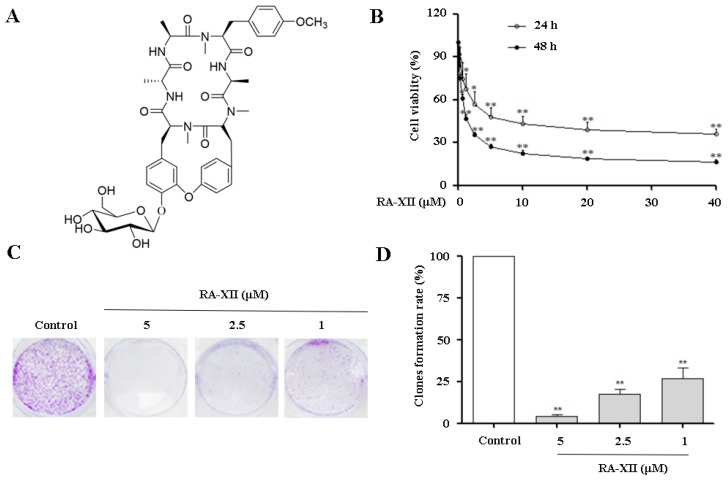
Effects of RA-XII on HepG2 cells growth and colony formation. (**A**) Chemical structure of RA-XII; (**B**) HepG2 cells were treated with RA-XII (0.5 to 40 μM) for 24 or 48 h. The cell viability was determined by sulforhodamine B (SRB) assay; (**C**) HepG2 cells were seeded in 6-well dishes, and after 48 h RA-XII treatment, they were reseeded and maintained for eight days to form colonies; (**D**) The number of clones was expressed by quantified analysis. The data are presented as mean ± SEM of three independent experiments (* *p* < 0.05, ** *p* < 0.01, vs. Control).

Currently, natural products are important resources for small molecules against cancer such as resveratrol [20], paclitaxel [21] and camptothecin [22]. Many traditional Chinese medicines exhibit anti-tumor effects and have been applied to the different stages of cancer treatment. Plant cyclopeptides have increasingly captured attention due to their anti-tumor activity [23,24,25]. RA-XII (Figure 1A), a unique natural cyclopeptide isolated from *Rubia yunnanensis*, exerts anti-cancer and anti-inflammatory activities [26,27]. In addition, RA-XII induced G1 arrest and inhibited tumor cell invasion and metastasis in breast cancer 4T1 cells [28]. However, the anti-tumor mechanism of RA-XII remains unclear in HepG2 liver cancer cells. In the present study, we firstly demonstrated that RA-XII inhibited protective autophagy and promoted apoptosis in HepG2 cells.

## 2. Results

### 2.1. RA-XII Inhibits Growth and Colony Formation in HepG2 Cells

Cell viability was assessed by sulforhodamine B (SRB) assay in HepG2 cells. As shown in Figure 1B, RA-XII inhibited the growth of HepG2 cells in a dose- and time-dependent manner with IC_50_ values of 6.51 μM and 1.34 μM for 24 or 48 h, respectively. Furthermore, to determine cell proliferation after RA-XII treatment, colony formation was measured. As illustrated in Figure 1C,D, RA-XII significantly decreased clonogenic survival of tumor cells in HepG2 cells.

### 2.2. RA-XII Induces Apoptosis in HepG2 Cells

**Figure 2:**
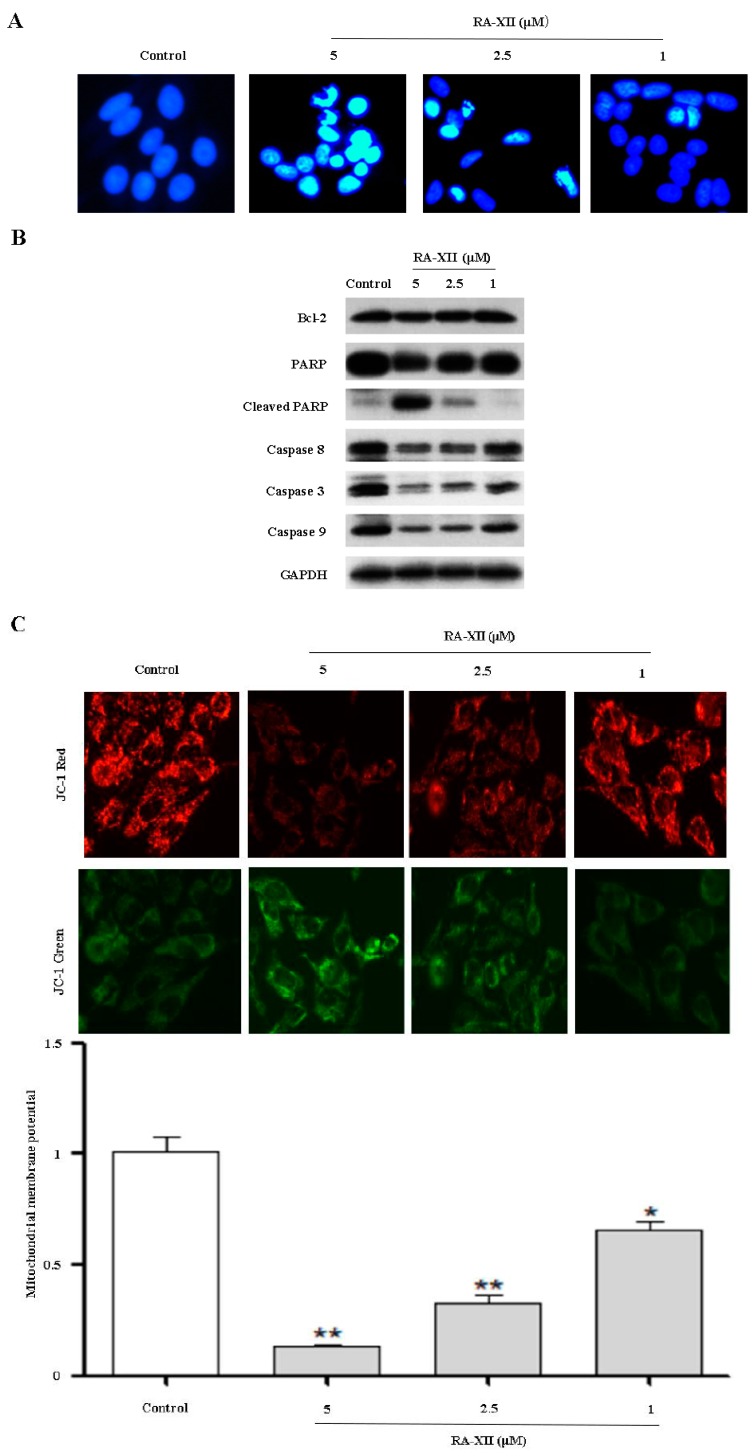

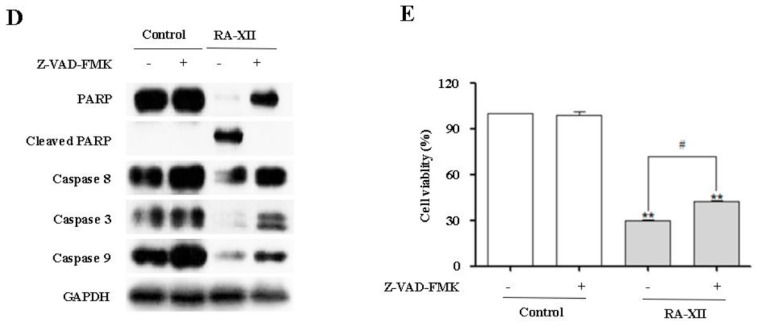
RA-XII induced apoptosis in HepG2 cells. (**A**) HepG2 cells were treated with RA-XII (5, 2.5, 1 μM) for 48 h. After fixation, staining with 4',6-diamidino-2-phenylindole (DAPI) and morphological characterization were analyzed by fluorescence microscope; (**B**) Effects of RA-XII on the expression of several classic marker of apoptosis. The levels of apoptosis-related protein (caspase 8, 3, 9, PARP, Bcl-2) were tested in total cell lysates from HepG2 cells treated RA-XII (5, 2.5, 1 μM) for 48 h by Western blot; (**C**) Cells were stained with JC-1 and photographed by fluorescence; (**D**) HepG2 cells were treated with or without RA-XII (5 μM) in the presence or absence of N-Benzyloxycarbonyl-Val-Ala-Asp(O-Me) fluoromethyl keton (Z-VAD-FMK) (25 μM), and analysis of apoptosis-related protein was performed by Western blot; (**E**) HepG2 cells were treated with the combination of RA-XII (5 μM) and Z-VAD-FMK (25 μM). The cell viability was determined by SRB. The data are presented as mean ± SEM of three independent experiments (** *p* < 0.01, vs. Control, ^#^ *p* < 0.05, vs. indicated treatment).

To determine the potential mechanism of RA-XII-inhibited proliferation in HepG2 cells, the ability of RA-XII to induce apoptosis was examined. As shown in Figure 2A, nuclear morphological changes of cells were photographed by DAPI using fluorescence microscopy. Morphologies of HepG2 cells were substantially changed by RA-XII treatment. RA-XII caused a dramatic increase in the number of apoptotic cells, as indicated by the condensed chromatin and fragmentation nuclei (shown as intense blue fluorescence). To further explore the underlying mechanism of RA-XII-induced apoptosis, the expressions of several classic apoptosis-related proteins were determined by Western blot. The expressions of caspase 3, 8 and 9 were dramatically reduced by RA-XII treatment while Cleaved-PARP (apoptosis marker protein) was up-regulated in a concentration-dependent manner in HepG2 cells (Figure 2B). Besides, the JC-1 assay revealed that RA-XII concentration-dependently caused reduction in the red fluorescence and elevation in the green fluorescence (Figure 2C), resulting in decreased mitochondrial membrane potential of HepG2 cells.

To elucidate the role of caspase activation in RA-XII-induced apoptosis, a pan-caspase inhibitor *N*-Benzyloxycarbonyl-Val-Ala-Asp (O-Me) fluoromethyl keton (Z-VAD-FMK) was applied to block caspase activation before RA-XII treatment. As expected, immunoblot data showed that Z-VAD-FMK increased the content of caspase 3, 8, 9, PARP and blocked Cleaved-PARP in the presence of RA-XII (Figure 2D). In line with the above results, RA-XII-treated HepG2 cells underwent cell death despite with or without Z-VAD-FMK (Figure 2E). RA-XII-induced cell death was only modestly rescued by Z-VAD-FMK in HepG2 cells.

### 2.3. RA-XII Suppresses Autophagy in HepG2 Cells

**Figure 3:**
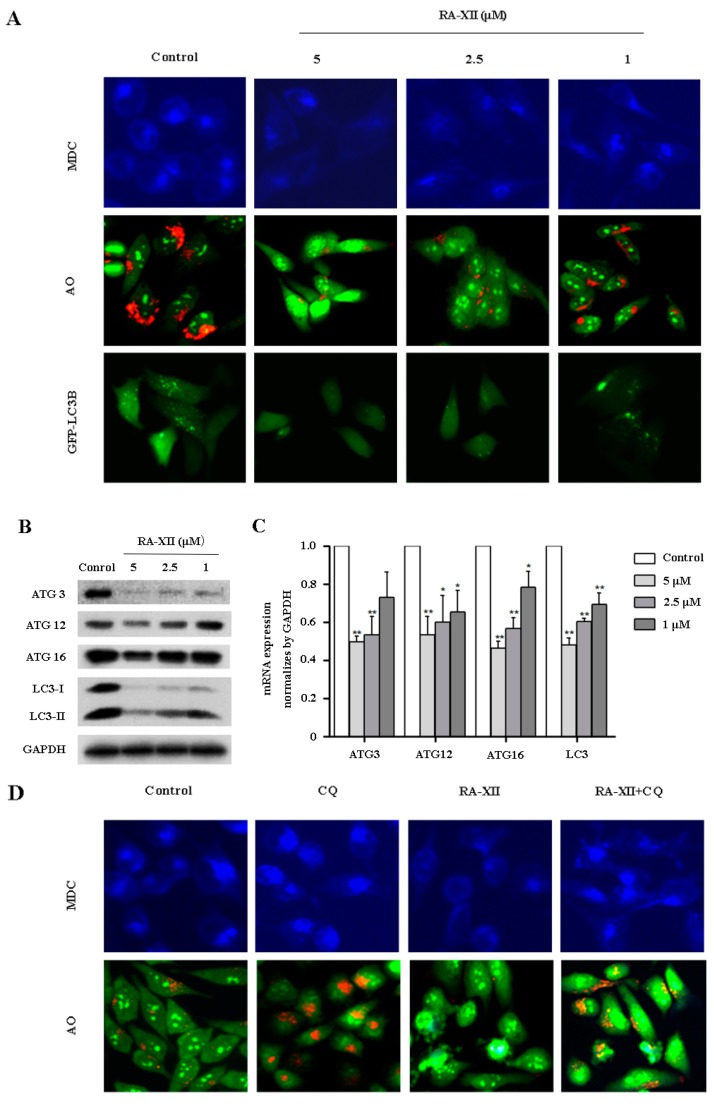

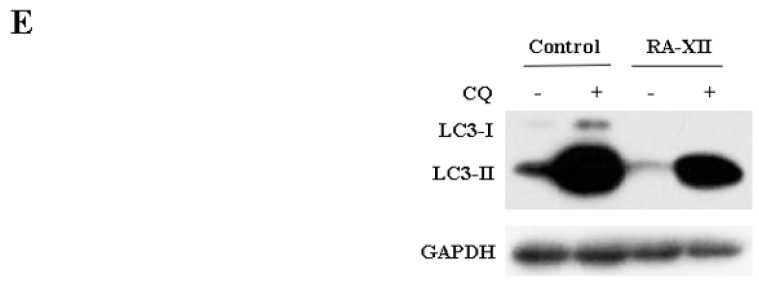
RA-XII suppressed autophagy in HepG2 cells. (**A**) HepG2 cells were treated with RA-XII (5, 2.5, 1 μM) for 48 h. Florescence microscopy analysis after (top panel) MDC staining, (middle panel) AO staining and (lower panel) transfection with GFP-LC3 plasmid; (**B**) Effects of RA-XII on the expression of several classic markers of autophagy. The levels of autophagy-related proteins (ATG3, 12, 16, LC3) in total cell lysates from RA-XII (5, 2.5, 1 μM)-treated HepG2 cells for 48 h were evaluated by Western blot; (**C**) The expressions of four indicated autophagy-related genes were measured by quantitative RT-PCR; (**D**) HepG2 cells were treated with or without RA-XII (5 μM) in the presence or absence of chloroquine (CQ, 25 μM) for 48 h. After stained with MDC and AO for 15 min at 37 °C, cells were imaged by florescence microscope; (**E**) HepG2 cells were treated with or without RA-XII (5 μM) in the presence or absence of CQ (25 μM) for 48 h, and the marker of autophagy LC3-II were analyzed by Western blot. The data are presented as mean ± SEM of three independent experiments (* *p* < 0.05, ** *p* < 0.01, vs. Control).

The accumulation of autophagic vesicles and formation of autolysosomes are two markers of autophagy, which could be stained by monodansylcadaverine (MDC) and acridineorange (AO), respectively. To determine whether RA-XII affected autophagy in HepG2 cells, MDC and AO staining were examined. As shown in Figure 3A, RA-XII treatment led to a significant reduction of MDC-stained autophagic vesicles in a concentration-dependent manner. Similar results were obtained from AO-stained orange-red autolysosomes (Figure 3A). Additionally, we tested LC3 to further verify autophagosome formation. Plasmids expressing green fluorescent protein GFP-LC3-fusion protein were transfected into HepG2 cells to label cytoplasmic autophagosomes. The number of GFP-LC3 fluorescent dots was dramatically decreased by RA-XII, indicative of the role of RA-XII in the suppression of autophagic vesicles production (Figure 3A). Meanwhile, we further examined an autophagosome marker LC3-II and autophagy-related protein ATG3, 12, 16 by Western blot and quantitative RT-PCR. As illustrated in Figure 3B,C, RA-XII dose-dependently triggered a significant reduction of LC3-II and ATG3, 12, 16 expression levels at both protein and mRNA levels in HepG2 cells.

Principally, reducing the autophagosome numbers can be associated either with inhibited autophagy initiation or excessive autophagosome degradation [29]. To clarify the effects of RA-XII, the autophagy flux was measured. Autophagy in the presence of the autophagy inhibitor chloroquine (CQ) was analyzed, which inhibits lysosome acidification and blocks downstream steps of autophagy. As shown in Figure 3D, RA-XII combined with CQ induced a significant increase of autophagosome compared with RA-XII treatment alone (Figure 3D). In addition, the influence of RA-XII on LC3-II levels with CQ was assessed. Expectedly, RA-XII reduced LC3 conversion which is induced by CQ in HepG2 cells (Figure 3E and Figure S1).

### 2.4. RA-XII Inhibits Protective Autophagy and Promotes Apoptosis in HepG2 Cells

**Figure 4:**
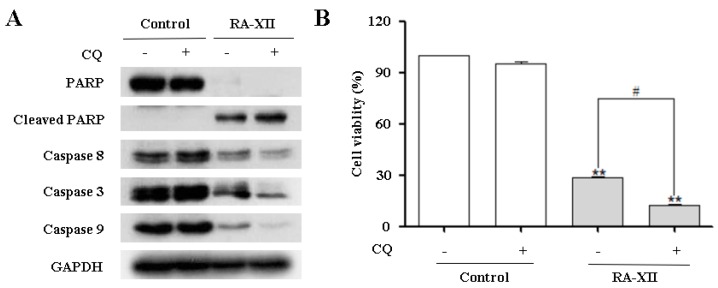
CQ contributes to RA-XII-induced apoptosis and cell death. (**A**) CQ promoted RA-XII-induced apoptosis. HepG2 cells were treated with or without RA-XII (5 μM) in the presence or absence of CQ (25 μM), and apoptosis-related proteins (caspase 8, 3, 9, PARP) were analyzed by Western blot; (**B**) HepG2 cells were treated with the combination of RA-XII (5 μM) and CQ (25 μM). The cell viability was determined by SRB. The data are presented as mean ± SEM of three independent experiments (** *p* < 0.01, vs. Control, # *p* < 0.05, vs. indicated treatment).

Recent studies suggest that autophagy may serve as a pro-survival or pro-death mechanism in different cellular contexts [10]. Besides, it has been reported that autophagy may facilitate cell survival in adverse microenvironments, and inhibition of autophagy may promote apoptosis induction [12]. In this study, RA-XII caused apoptosis and inhibited autophagy (Figure 2 and Figure 3). In view of the crucial role of RA-XII on the association of apoptosis and autophagy, how autophagy inhibition affected RA-XII-induced apoptosis and cytotoxicity was investigated. Combinational treatment with RA-XII and CQ obviously increased Cleaved PARP and decreased caspase 8, 9 and 3 compared to RA-XII alone, indicating the stimulating effects of apoptosis (Figure 4A). Furthermore, RA-XII-mediated cell death was strikingly enhanced in the presence of CQ (Figure 4B).

### 2.5. Akt/AMPK-mTOR Signaling Pathways are Involved in RA-XII-Inhibited Protective Autophagy in HepG2 Cells

**Figure 5:**
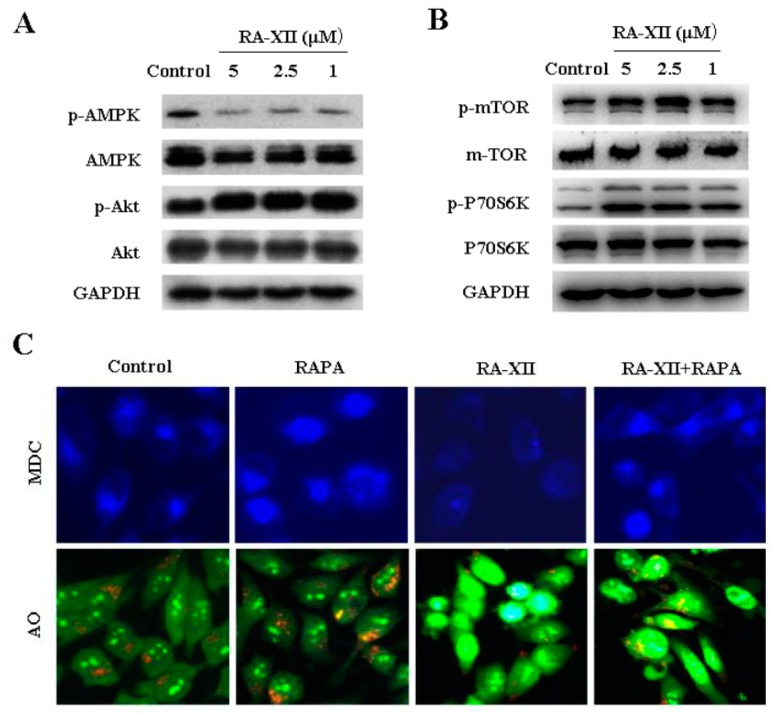

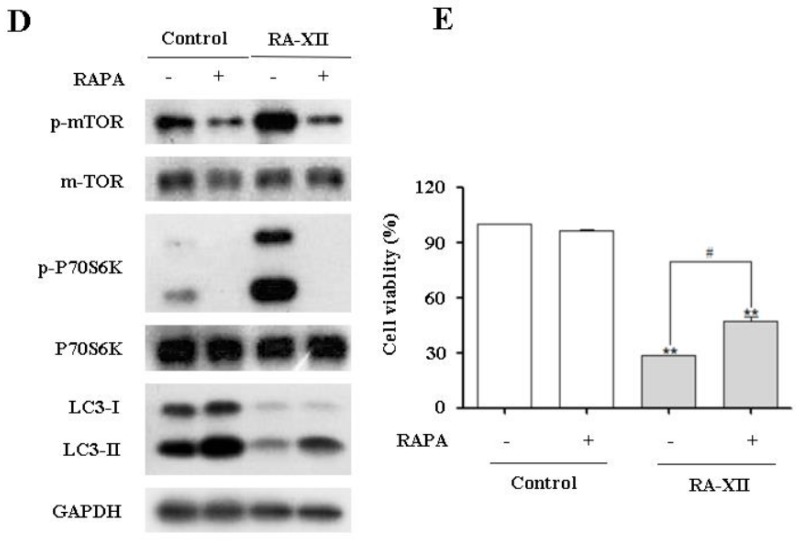
RA-XII down-regulated AMPK and up-regulated Akt-mTOR-P70S6K pathways in HepG2 cells. (**A**,**B**) AMPK, Akt, mTOR, and their phosphorylated forms were analyzed by Western blot; (**C**) HepG2 cells were treated with or without RA-XII (5 μM) in the presence or absence of rapamycin (RAPA) (1 μM). After stained with MDC and AO for 15 min at 37 °C, cells were imaged under a florescence microscope; (**D**) HepG2 cells were treated with or without RA-XII (5 μM) in the presence or absence of RAPA (1 μM), and mTOR, P70S6K and their phosphorylated forms were analyzed by Western blot; (**E**) HepG2 cells were treated with RA-XII (5 μM) combined with RAPA (1 μM). The cell viability was determined by SRB. The data are presented as mean ± SEM of three independent experiments (** *p* < 0.01, vs. Control, ^#^ *p* < 0.05, vs. indicated treatment).

Increasing evidence indicates that mTOR plays a pivotal role in the control of autophagy, which integrates input information from multiple upstream signal transduction pathways and negatively regulates autophagy. To obtain some insights into the mechanism of RA-XII-inhibited protective autophagy in HepG2 cells, the activation status of autophagy-related Akt and AMPK/mTOR/P70S6K signaling pathways were analyzed. Phosphor-Akt, phosphor-AMPK, phosphor-mTOR and phosphor-P70S6K (ribosomal protein S6 kinase, 70 kDa, polypeptide 1) were detected. As shown in Figure 5A, RA-XII reduced phosphor-AMPK, while phosphor-mTOR and phosphor-P70S6K were elevated by RA-XII treatment (Figure 5A,B).

To further elucidate the role of mTOR activation in RA-XII–inhibited protective autophagy, we blocked the mTOR signaling with a special mTOR inhibitor rapamycin (RAPA). Treatment with RA-XII in the presence of RAPA increased MDC- and AO- stained autophagic vesicles compared with RA-XII-treated cells alone (Figure 5C). HepG2 cells were pre-treated by RAPA before treating with RA-XII. The results showed that RAPA markedly attenuated RA-XII-mediated mTOR phosphorylation and P70S6K phosphorylation in HepG2 cells (Figure 5D). Moreover, RAPA could rescue RA-XII-induced cell death, suggesting that the role of mTOR in the regulation of HepG2 cells survive (Figure 5E).

## 3. Discussion

**Figure 6:**
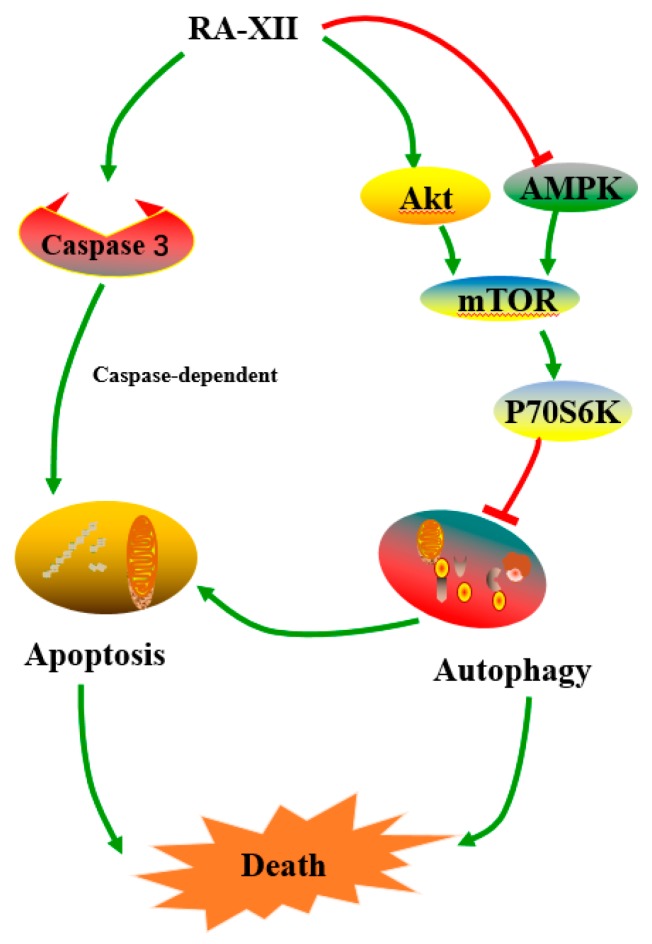
Proposed mechanism of RA-XII on apoptosis and autophagy.

The present study was designed to clarify the underlying anti-tumor mechanisms of RA-XII in HepG2 cells. The results indicated that RA-XII inhibited the proliferation of HepG2 cells in a concentration-dependent manner and suppressed protective autophagy and promoted apoptosis. RA-XII treatment substantially reduced both autophagosomes and autophagic flux through activation of mTOR signaling pathway in HepG2 cells (Figure 6).

In this study, we found that RA-XII displayed cytotoxicity activity in HepG2 cells (Figure 1B). Notably, RA-XII inhibited HepG2 cells colony formation ability (Figure 1C,D). The anti-tumor activity is partially due to the induction of the apoptosis program (Figure 2A). Apoptosis is commonly thought to be the main anti-cancer mechanism of chemotherapy and can be triggered by various anti-tumor stimulation. Once apoptosis is triggered, caspases including initiator caspases and effector caspases are activated through proteolytic cleavage. The active effector caspases then activate downstream molecules, such as PARP, and proteolytically degrade plenty of intracellular proteins to execute apoptosis cell death program. In this paper, the typical apoptotic changes were observed in RA-XII-treated HepG2 cells. RA-XII significantly led to activation of caspase 3, 8, 9 and PARP, and downregulation of Bcl-2 (Figure 2B), accompanied by a decrease of mitochondrial membrane potential (Figure 2C). However, RA-XII-induced cell death was only modestly rescued by Z-VAD-FMK in HepG2 cells (Figure 2D,E). Together, these results indicated that cell death triggered by RA-XII might be partially due to apoptosis.

Autophagy, another cell death program, is associated with cancer cell death [11]. Under extreme conditions, autophagy served as a self-protection mechanism supplying energy and nutrients for cancer cells to survive, which can lead to tumor progression and therapeutic resistance. Additionally, the process of tumor cells metastasis may particularly rely on autophagy. Inhibition of autophagy may enhance the sensitivity of cancer cells to cytotoxic drugs [13]. Given that, many autophagy inhibitors have been identified and were used in cancer treatment because they can target tumor cells in areas of low oxygen [30]. Phase I clinical evaluation of hydroxychloroquine (HCQ) combination with other anti-tumor drugs in cancer patients has been performed [31,32]. Currently, inhibition of the autophagic pathway is regarded as a promising new strategy for cancer treatment. Therefore, a novel inhibitor of autophagy with a lower toxicity and better therapeutic index is needed.

RA-XII was identified as a potent inhibitor of autophagy, which was tested with several different assays including autophagosome and autophagic flux. Results from MDC and AO staining indicated that RA-XII significantly reduced the production of autophagic vesicles and the number of GFP-LC3 fluorescent dots dramatically decreased by RA-XII (Figure 3A). Meanwhile, RA-XII suppressed a concentration-dependent conversion LC3-I to LC3-II detected by immunoblotting and inhibited ATG3, 12, 16 expression levels at both protein and mRNA levels (Figure 3B,C). All of these data indicated that RA-XII effectively decreased the autophagosome number and inhibited autophagic flux in HepG2 cells. Currently, autophagy inhibitors can be divided into two categories: early-stage inhibitors and late-stage inhibitors. Early-stage autophagy inhibitors, including wortmannin [33], LY294002 [33], and 3-MA, mainly block the formation of autophagosomes through the inhibition of class III PtdIns 3-kinases. The late-stage autophagy inhibitors, such as CQ, HCQ, bafilomycin A1, and lysosomal protease inhibitors, exert suppressive effects on downstream of autophagosome formation, including inhibition of autophagosome and lysosome fusion and/or blocking degradation of autophagic cargo inside autolysosomes [14]. As expected, RA-XII significantly reduced LC3-II levels in the presence of CQ compared to CQ alone (Figure 3E). These data provided evidence that RA-XII belonged to the early-stage autophagy inhibitor.

Promoting apoptosis has been a critical strategy for anti-tumor therapy during the past several decades. However, recent study has shown that even when apoptosis is blocked, activation of autophagy is still indispensable for tumor cells to survive metabolic stresses [12]. These findings highlight the essential role of autophagy in tumor cell survival under stresses. However, the exact role of autophagy in cancer treatment, and whether it protects cells from cytotoxic effects of anti-cancer drugs by blocking apoptosis or kills cells as an alternate pathway of cell death is still controversial. Herein, the results revealed that RA-XII could inhibit autophagy accompanied by apoptosis in HepG2 cells at the same concentration range. Besides, the inhibition of autophagy by CQ potentiated RA-XII-induced apoptosis and increased the cytotoxic effects of RA-XII in HepG2 cells (Figure 4A,B). Meanwhile, autophagy inducer RAPA increased cell viability (Figure 5E). Collectively, the results implied that RA-XII inhibited protective autophagy to promote apoptosis. It is worth mentioning that Z-VAD-FMK could not obviously rescue RA-XII-induced cell death, and a significant reduction of AO-stained orange-red acidic vesicle was observed upon RA-XII-treated cells, which indicated that RA-XII might induce caspase-independent cell death, such as lysosomal mediated cell death [34].

mTOR is a serine or threonine protein kinase that regulates growth, proliferation, survival, protein synthesis and is a target for anti-cancer agents [35]. mTOR is sensitive to inhibition by RAPA [36]. Inactivation of mTOR by RAPA stimulates autophagy in the presence of nutrients, suggesting that mTOR downregulates autophagy. The phosphoinositide 3-kinase (PI3K)-activated serine/threonine kinase Akt can positively phosphorylate the mTOR and inhibit autophagy initiation through blocking the expression and function of autophagy-related protein [37,38,39]. Moreover, emerging evidence demonstrates that another main mTOR regulator, AMPK, the fuel sensor of mammalian cells, suppresses mTOR activity in the early stage of autophagy [40,41,42]. In this study, RA-XII significantly increased the level of p-Akt, p-mTOR and p-P70S6K and reduced the level of p-AMPK (Figure 5A,B). This suggested that RA-XII led to the activation of Akt/mTOR pathway and inhibited the AMPK pathway, and further indicated that RA-XII inhibited protective autophagy through activation of mTOR pathway.

mTOR signaling pathway couples energy and nutrient abundance to the execution of cell growth and division, due to the ability of mTOR to simultaneously sense energy, nutrients and stress in metazoans growth factors [43]. The role of mTOR is to activate translation of proteins. However, the role of mTOR in cancer shows two faces [44]. Some reports showed that mTOR signaling is an important pathway for cell growth and proliferation [43]. However, other reports showed that mTOR activation induces tumor suppressors that inhibited cancer cell proliferation [45]. Meanwhile, constitutive activation of mTOR can lead to suppression of autophagy and enhanced susceptibility to stress-mediated cell death [46,47]. A positive or negative role for mTOR in tumor growth clearly depends on the complicated environment. This study revealed that activation of the mTOR signaling pathway contributed to RA-XII-induced tumor cells death.

Liver cancer is considered to be an untreatable solid tumor. After long-term therapy, a large number of patients of advanced liver cancer eventually become treatment-resistant or there is a recurrence due to the persistence of the remainder of solid tumor tissue. The remainder of tumor cells can progress via autophagy, which leads to recurrence after radiotherapy or chemotherapy stops [48]. Thus, autophagy inhibition is particularly important in this process for the treatment of liver cancer. Although there are several clinical trials of autophagy suppression to treat cancer, liver cancer is not involved. The data from the present study explored that, for the first time, RA-XII is a novel natural type of autophagy inhibitor, which has potential anti-tumor effects and can be used as a drug or the combination therapy to kill liver cancer cells.

## 4. Materials and Methods

### 4.1. Cell Culture

HepG2 cells were obtained from the Cell Bank of Chinese Academy of Sciences (Shanghai, China) and cultured at 37 °C in a 5% (*v*/*v*) CO_2_ atmosphere. Cells were maintained with DMEM medium supplemented with 10% fetal bovine serum and antibiotics (100 U/mL penicillin and 100 mg/mL streptomycin).

### 4.2. Reagents and Antibodies

RA-XII (purity ≥ 99%) was prepared in our laboratory, which was identified by MS and NMR spectroscopies [27]. RA-XII was dissolved at the concentration of 20 mM in DMSO as a stock solution, stored at −20 °C, and diluted with medium for each experiment. DMEM, fetal bovine serum and lipofectamine 2000 were purchased from Life Technologies (Burlington, ON, Canada). Chloroquine (Sigma, St. Louis, MO, USA) was dissolved in phosphate-buffered saline. Rapamycin (Sigma, St. Louis, MO, USA), Z-VAD-FMK (Sigma, St. Louis, MO, USA), MDC (Sigma, St. Louis, MO, USA) and AO (Sigma, St. Louis, MO, USA) were dissolved in DMSO (<0.1%). GFP-LC3B plasmid was purchased from Miaolingbio (Wuhan, China). The antibodies as follows: LC3-II, ATG3, 12, 16, Bcl-2, p-AMPK, p-Akt, p-mTOR, and p-P70S6K (Cell Signaling Technology, Danvers, MA, USA); and GAPDH, Akt, AMPK, mTOR, P70S6K, caspase 3, 8, 9 and PARP (Proteintech, Chicago, IL, USA).

### 4.3. Cell Viability Assay

Cell viability was determined via sulforhodamine B (SRB) assay. HepG2 cells were seeded in 96-well plates at a density of 1 × 10^4^ cells and incubated for 24 or 48 h. After treatment with RA-XII, 100 μL of 10% TCA was added to each well and fixed in for 1 h. After washing three times with water, 100 μL of 4% SRB was added to each well and stained 15 min. Then washed three times with 1% TCA, stained cells were dissolved with 10 mM unbuffered Trisbase (pH = 10.5) and were measured the absorbance at 540 nm using a microtitre plate reader (Bio-Rad, Hercules, CA, USA).

### 4.4. Clonogenic Survival Assay

HepG2 cells (500 cells/well) were seeded with culture medium in 6-well plates and incubated at 37 °C for 24 h. Cells were treated with RA-XII (5, 2.5, 1 μM) for another 48 h. Then, cells were washed with PBS before being incubated in fresh medium and maintained for 8 days at 37 °C. Finally, the cultures were fixed with methanol and stained with trypan blue. The colony numbers with >50 cells were counted under an inverted microscope.

### 4.5. Mitochondrial Membrane Potential (JC-1) Assay

The mitochondrial membrane potential disruption was measured using JC-1 (Sigma, St. Louis, MO, USA) probe. HepG2 cells were treated with vehicle or desired concentrations of RA-XII for 48 h. Cells were incubated with JC-1 (5,5′,6,6′-tetrachloro-1,1′,3,3′-tetraethylbenzimidazol-carbocyanine iodide, 10 μg/mL) for 20 min at 37 °C. The staining buffer was removed and cells were washed with ice-cold PBS twice. Two milliliter DMEM was added to each well. JC-1 loaded cells were observed with a fluorescence microscope (Leica, Wetzlar, Germany).

### 4.6. GFP-LC3B Plasmid Transfection

HepG2 cells were seeded at 8 × 10^5^ per well in a 24-well plate and allowed to adhere for 24 h. Transfection with GFP-LC3 plasmid was then carried out using lipofectamine 2000 according to the manufacturer’s instructions. After 24 h, cells were treated for 48 h with RA-XII or with controls (blank and DMSO). Cells were then washed three times by PBS and analyzed in a fluorescence microscope.

### 4.7. DAPI Staining

Morphological changes of HepG2 cells were determined by DAPI staining. HepG2 cells were treated with vehicle or desired concentrations of RA-XII for 48 h. After washing with PBS, cells were fixed with methanol/acetone (1:1) for 5 min at room temperature. The fixed cells were then washed with PBS and permeabilized with 0.1% TritonX-100 for 10 min prior to staining with DAPI (1:2000 dilution, in PBS) for 10 min. Cells were washed with PBS and mounted. Images of DAPI fluorescence were collected using a Nikon phase-fluorescence microscope. Moderately fluorescent and round nuclei were considered normal. Bright and condensed/fragmented nuclei were regarded as apoptotic.

### 4.8. Staining of Autophagic Vacuoles by Monodansylcadaverine (MDC) and Acridine Orange (AO)

Fluorescent probe MDC and AO are selective markers for acidic vesicular organelles to evaluate autophagy. HepG2 cells were grown on coverslips and treated with RA-XII (5, 2.5, 1 μM) for 48 h. For MDC staining, cells were then exposed to MDC (50 μM) at 37 °C for 15 min in the dark. For AO staining, cells were incubated with AO (1 μM) for 15 min. After washing with PBS, cells were visualized under fluorescence microscope.

### 4.9. Immunoblotting Assay

After treatment with RA-XII at desired concentrations, HepG2 cells were lysed with RIPA lysis buffer containing PMSF. Cell lysates were centrifuged at 12,000× *g* for 30 min. Equal amounts of the protein were resolved by SDS–PAGE and transferred into a nitrocellulose membrane. The membranes were blocked with 5% non-fat dry milk in TBS for 1 h at room temperature and then incubated with primary antibodies at 4 °C overnight. Membranes were washed and treated with appropriate secondary antibodies for 1 h at room temperature. The immunocomplexes were detected with the enhanced chemiluminescence plus kit (Pierce, Waltham, MA, USA).

### 4.10. Quantitative Real-Time PCR

Total RNA was extracted with Trizol reagent (Invitrogen, Carlsbad, CA, USA) following the manufacturer’s protocol. The RT-PCR was performed with TAKARA (Otsu, Japan) according to the manufacturer’s instructions. To detect the mRNA levels of ATG3, ATG12, ATG16 and LC3, primers used were as follows: ATG3 forward, 5′-TCACAACACAGGTATTACAGG-3′, reverse, 5′-TCACCGCCAGCATCAG-3′; ATG12 forward, 5′-TCTATGAGTGTTTTGGCAGTG-3′, reverse, 5′-ATCACATCTGTTAAGTCTCTTGC-3′; ATG16 forward, 5′-TCCAGGAGGCGGCAAG-3′, reverse, 5′-ATCAGAAGTTTCATCCACAATG-3′; LC3 forward, 5′-CTGAGATTGGTGTGGAGACG-3′; reverse, 5′-CGGTGATAATAGAACGATACAAGG-3′; GAPDH forward, 5′-GGGAAGCTTGTCATCAATGG-3′, reverse, 5′-CATCGCCCCACTTGATTTTG-3′. The GAPDH was used as an internal control.

### 4.11. Statistical Analysis

Statistical analysis was conducted using GraphPad Prism V software. Data were analyzed using ANOVA and unpaired student’s *t*-test methodologies. Cases in which *p* < 0.05 or *p* < 0.01 were considered statistically significant.

## 5. Conclusions

In conclusion, RA-XII significantly inhibited the HepG2 cells growth in a dose- and time-dependent manner. RA-XII promoted apoptosis and inhibited protective autophagy through AMPK/mTOR/P70S6K pathways in HepG2 cells (Figure 6). These findings suggest that RA-XII may potentially be a candidate as an autophagy inhibitor agent of further therapy for liver cancer.

## Supplementary Materials

Supplementary Materials are available online, Figure S1: Statistical graph of Figure 3E.
