# Supplementary material for: Natural Cyclopeptide RA-XII, a New Autophagy Inhibitor, Suppresses Protective Autophagy for Enhancing Apoptosis through AMPK/mTOR/P70S6K Pathways in HepG2 Cells
Source: Molecules. 2017 Nov 11;22(11):1934. doi: 10.3390/molecules22111934 (PMC6150396; doi:10.3390/molecules22111934)
Supplement: Supplementary file 1 [file molecules-22-01934-s001.pdf]

Article

Natural cyclopeptide RA-XII, a new autophagy inhibitor, suppresses protective autophagy for enhancing apoptosis through AMPK/mTOR/P70S6K pathways in HepG2 cells

Lihua Song[1,†], Zhe Wang[1,†], Yurong Wang[1], Di Guo[1], Jianhong Yang[2], Lijuan Chen[2] and Ninghua Tan[1,3,*]

[1] School of Traditional Chinese Pharmacy, China Pharmaceutical University, 639 Longmian Avenue, Nanjing 211198, China; songlihua4835@163.com(L.S.); wangzhe153807105@163.com(Z.W.); yurong1987213@163.com(Y.W.); guodi33@163.com(D.G.)

[2] State Key Laboratory of Biotherapy, Sichuan University, and Collaborative Innovation Center for Biotherapy, Chengdu 610041, China; yjh0742043024@hotmail.com(J.Y.); ljchen@scu.edu.cn(L.C.)

[3] State Key Laboratory of Phytochemistry and Plant Resources in West China, Kunming Institute of Botany, Chinese Academy of Sciences, Kunming 650201, China

* Correspondence: nhtan@cpu.edu.cn; Tel.: +86-25-84155947

† These authors contributed equally to this work.

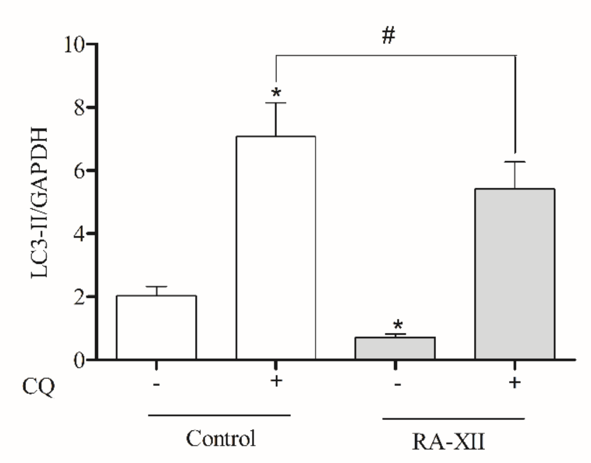

Figure S1. Statistical graph of Figure 3E. HepG2 cells were treated with or without RA-XII (5 μM) in the presence or absence of CQ (25 μM) for 48 h, and the marker of autophagy LC3-II were analyzed by Western blot. These bands were quantified by statistical graph. The data are presented as mean ± SEM of three independent experiments. (*$p < 0.05$, vs Control, #$p < 0.05$, vs indicated treatment.)
